# Expression Divergence as an Evolutionary Alternative Mechanism Adopted by Two Rice Subspecies Against Rice Blast Infection

**DOI:** 10.1186/s12284-019-0270-5

**Published:** 2019-03-01

**Authors:** Zhenhui Zhong, Lianyu Lin, Meilian Chen, Lili Lin, Xiaofeng Chen, Yahong Lin, Xi Chen, Zonghua Wang, Justice Norvienyeku, Huakun Zheng

**Affiliations:** 1 State Key Laboratory of Ecological Pest Control for Fujian and Taiwan Crops, College of Plant Protection, Fujian Agriculture and Forestry University, Fuzhou, 350002 China; 2 Fujian-Taiwan Joint Center for Ecological Control of Crop Pests, Fujian Agriculture and Forestry University, Fuzhou, 350002 China; 3 College of Life Science, Fujian Agriculture and Forestry University, Fuzhou, 350002 China; 4 Institute of Oceanography, Minjiang University, Fuzhou, 350108 China

**Keywords:** Expression divergence, Rice blast disease, Pathogen response, Rice divergence

## Abstract

**Background:**

Rice (*Oryza sativa* L.) is one of the most important crops that serves as staple food for ~ 50% of the human population worldwide. Some important agronomic traits that allow rice to cope with numerous abiotic and biotic stresses have been selected and fixed during domestication. Knowledge on how expression divergence of genes gradually contributes to phenotypic differentiation in response to biotic stress and their contribution to rice population speciation is still limited.

**Results:**

Here, we explored gene expression divergence between a *japonica* rice cultivar Nipponbare and an *indica* rice cultivar 93–11 in response to invasion by the filamentous ascomycete fungus *Magnaporthe oryzae* (*Pyricularia oryzae*), a plant pathogen that causes significant loss to rice production worldwide. We investigated differentially expressed genes in the two cultivars and observed that evolutionarily conserved orthologous genes showed highly variable expression patterns under rice blast infection. Analysis of promoter region of these differentially expressed orthologous genes revealed the existence of *cis*-regulatory elements associated with the differentiated expression pattern of these genes in the two rice cultivars. Further comparison of these regions in global rice population indicated their fixation and close relationship with rice population divergence.

**Conclusion:**

We proposed that variation in the expression patterns of these orthologous genes mediated by *cis*-regulatory elements in the two rice cultivars, may constitute an alternative evolutionary mechanism that distinguishes these two genetically and ecologically divergent rice cultivars in response to *M. oryzae* infection.

**Electronic supplementary material:**

The online version of this article (10.1186/s12284-019-0270-5) contains supplementary material, which is available to authorized users.

## Background

Expressional divergence mediated by variation in *cis*-elements has long been considered as an important force in phenotypic evolution (Siepel and Arbiza 2014). Generally, *cis*-elements are localized upstream of the coding sequence and manipulate the expression pattern of genes by altering the binding of transcription factors required for gene expression (Wray 2007). Accumulated data indicated that changes in *cis*-elements could lead to phenotypic variation and consequently cause phenotypic evolution within or between species. For example, the homologous proteins in humans and chimpanzees share more than 99.5% identity, but the two species differ from each other in many phenotypes (King and Wilson 1975), which was later turned out to be the consequences of divergent expression caused by variation in *cis*-elements associated with their coding genes (Siepel and Arbiza 2014). Similar mechanism was also identified in other species, such as *Arabidopsis* (Arsovski et al. 2015), sorghum (Jiang et al. 2013) and *Drosophila (*Osada et al. 2006; Rifkin et al. 2003). However, most of these studies were focused on the developmental phenotype, and how does the *cis*-elements mediated expressional divergence contributes to phenotypic differentiation in response to diverse stresses remains largely unknown.

Rice (*Oryza sativa* L.) is one of the most important crops that serves as staple food for more than half of the world’s population. Over the years, rice has evolved into several subspecies as a result of purposeful breeding and intensive domestication. These different rice subspecies are often endowed with distinct agronomic traits, such as larger grain size, superior grain quality as well as biotic and abiotic stress tolerance (Wang et al. 1998). The speciation of rice species is greatly influenced by human activities. Recent rice population genetic studies demonstrated that the two most widely distributed rice subspecies *japonica* Group and *indica* Group evolved from a common ancestor, *Oryza rufipogon* several thousand years ago (Huang et al. 2012, Wang et al. 2018b, Xie et al. 2015). Additional rice population studies further showed that environmental factors, especially temperature, exerted great influence on the genomic characteristics of *japonica* and *indica* rice cultivars (Huang et al. 2012). Many of the domestication related genes have been identified as key regulators of important agronomic traits, such as seed shattering, pericarp color, amylose content, as well as grain number or size in rice, and these genes have shown significant variation between rice subspecies. For instance, a major shattering QTL, qSH1, has been identified in all temperate *japonica* rice but absent in tropical *japonica* and *indica* rice (Konishi et al. 2006). Some differentially expressed genes in response to various signals or different life stages which lead to distinct features have been fixed during the domestication history of rice (Jung et al. 2008; Liu et al. 2010a; Lu et al. 2010). Recent studies revealed SNPs or InDels at promoter regions between rice subspecies that leads to dramatic phenotypic differentiation in rice blast resistance, grain yield and nitrogen utilization (Bai et al. 2017; Huang et al. 2018; Li et al. 2017; Wang et al. 2018a). However, the origin and genomic foundations related with SNPs or InDels mediated variation on gene expression still need to be uncovered.

In an attempt to control the development of rice blast disease, caused by the filamentous ascomycete fungus *Magnaporthe oryzae* (*Pyricularia oryzae*), which causes significant yield losses in rice producing regions across the world, hundreds of resistance related genes have been domesticated along with human rice breeding history (Dean et al. 2012; Ebbole 2007; Li et al. 2017; Liu et al. 2010b). Similar to other distinctly manipulated agronomic traits, *japonica* and *indica* cultivars exhibited different degrees of resistance against *M. oryzae* (Bhuiyan et al. 2007; Gallet et al. 2016). Our recent results implied *japonica* and *indica* cultivation may shape population divergence of *M. oryze* (Zhong et al. 2018). However, it’s still not clear how expression level variation of rice blast infection responsive genes shapes the population speciation of rice.

Here, we profiled a *japonica* Group cultivar (Nipponbare, NPB) and an *indica* Group cultivar (93–11) in response to the fungal pathogen infection, by mainly focusing on the performance of orthologous genes. Our analysis showed that the distinct expression patterns exhibited by orthologous genes identified in the two rice cultivars corresponded with the high sequence diversity prevailing in the promoter region. Additionally, we further investigated genetic divergence of identified sequences in 3,010 rice cultivars and found that these regions exhibited higher fixation index (Fst) between *japonica* and *indica* rice populations, which indicates that these regions undergone strong selection during rice population divergence.

## Methods

### Rice Cultivation and Inoculation

*Magnaporthe oryzae* isolates Guy11 and FJ81278 were used for inoculating rice. The conidia harvested from 10-day-old colonies were cultured on rice-bran agar medium (2% rice-polish, 1.5% agar, and pH 6.5) at 26 °C under constant light. Conidia suspensions were adjusted to 1.5–2.0 × 10^5^ conidia/mL in solution containing 0.02% Tween before using it to inoculate four-week-old rice seedlings (*Oryza sativa* L.) cultivar Nipponbare (NPB, *japonica*) and 93–11 (*indica*) along with control respective groups (rice seedlings sprayed with solution containing only 0.02% Tween). To avoid the influence of dark treatment after inoculation, both inoculated rice and control groups were incubated in the dark, humid chamber at 25 °C for 24 h before transferring them into another humid chamber with 12 h photoperiod or sample collection. Plant tissues were collected at 24 hpi (collect both mock samples and samples inoculated by isolates for 24 h) and 48 hpi. All the samples were immediately frozen in liquid nitrogen after collection and stored at − 80 °C. The experimental samples were generated from three biological repeats.

### RNA Extraction, Sequencing Library Construction and Illumina Sequencing

Total RNA was extracted with RNAprep pure Plant Kit (Tiangen, Beijing) according to the manufacturer’s instructions. RNA integrity and quantity was verified using a 2100 Bioanalyzer (Agilent Technologies, Santa Clara, CA) according to the manufacturer’s instructions. The cDNA library was sequenced using the Illumina sequencing platform (IlluminaHiSeq™ 2000) with 125 bp pair-end reads length and 280 bp insert size by Gene Denovo Co. (Guangzhou, China). An in-house Perl program was used to select clean reads by removing adaptor sequences, low quality sequences (reads with more than 50% of base quality lower than 20) and reads with more than 5% N bases.

### Reads Alignment and Normalization of Gene Expression Levels

The reference genome for RNA seq analysis is Nipponbare genome (*Oryza sativa japonica*: http://plants.ensembl.org/Oryza_sativa/Info/Index). Cleaned short reads were aligned to all exon sequence by Bowtie2 (Langmead and Salzberg 2012), and expression abundance were calculated by RSEM with default parameters (Li and Dewey 2011). Gene expression level obtained from RNA-Seq analysis was validated by qRT-PCR for 10 DEGs. Primers used in the study were as shown in Additional file 1: Table S2.

### Differentially Expressed Genes (DEGs) and Function Enrichment Analyses

To reduce false positive of differential expression, all isoforms with less than 200 reads count in total were regarded as low-expressed genes and have been removed in the following analysis. Differential expression analysis was conducted with edgeR (Robinson et al. 2010). False discovery rate (FDR) was used to control the *P*-value and a threshold of FDR < 0.05 and |Log2FC| > 1 was used in determining significant differences in the expression level between samples. Gene Ontology Consortium (http://geneontology.org/) was used for functional enrichment analysis of differentially expressed genes (Ashburner et al. 2000).

### Identification of Orthologous Genes, Collinearity Analysis and Motif Enrichment

Orthologous genes from NPB and 93–11 were identified by NCBI-BLASTN with 500 xdrop-gap parameters. To reduce the redundancy of orthologous genes obtained from BLASTN, 2 kb upstream sequence was also included for searching the best hit that shares the most similar coding and flanking sequence. MCScanX was used to identify the collinearity between NPB and 93–11 with default parameters (Wang et al. 2012). Genome collinearity was visualized with Circos (Krzywinski et al. 2009). To analyze orthologous genes in other rice genome, we have searched orthologous genes in 3 *indica* rice (Zhenshan 97, Minghui 63 (Zhang et al. 2016), Shuhui 498 (Du et al. 2017)) and 2 *japonica* rice (HEG4, A123 (Lu et al. 2017)) with NCBI-BLASTN.

Motif enrichment was performed by using the HOMER findMotifs tool with default parameters (Heinz et al. 2010). Selection of input sequences and background sequences for motif enrichment are as shown in Fig. 3b and c.

### Nucleotide Diversity Calculation and Population Genomic Data Analysis

2 kb upstream sequence of orthologs was extracted and Needle was used for pair-wise sequence alignment (Rice et al. 2000). DnaSP was used for nucleotide diversity calculation (Rozas et al. 2017). SNPs data of 1,770 *indica* and 850 *japonica* rice were obtained from Rice SNP-Seek Database (http://snp-seek.irri.org/) for population genomics analysis (Wang et al. 2018b). Samtools and Vcftools was used to calculate the fixation index (Fst) of different orthologs (Danecek et al. 2011; Li et al. 2009).

## Results

### RNA-Seq Sequencing of NPB and 93–11 in Response to *M. oryzae* Infection

To investigate the expression divergence between *japonica* and *indica* rice groups under *M. oryzae* infection, we performed comparative transcriptome analysis between NPB and 93–11 challenged with two *M. oryzae* strains, Guy11 and FJ81278, respectively. NPB and 93–11 were used as representative rice cultivars of *japonica* and *indica,* respectively, in this study, since the genome sequences of these two rice cultivars are well described in previous studies (Goff et al. 2002; Yu et al. 2002). While FJ81278 (FJ87) belongs to the *indica*-infecting lineage (clade3), Guy11 (GY11) belongs to a lineage that consisted both the *indica*-infecting and *japonica*-infecting population (Bao et al. 2017; Zhong et al. 2018). Infection assay of NPB/93–11 with GY11/FJ87 showed that both isolates can cause susceptible reaction on the two rice cultivars with slightly different aggressiveness at 18, 24, 36 and 48 h post inoculation (hpi) (Additional file 1: Figure S1). The workflow portraying sample inoculation and collection is shown in Additional file 1: Figure S2. Rice samples treated with or without *M. oryzae* spore solutions were collected at 24 hpi and 48 hpi respectively. In total, 402 million and 434 million of 125 bp paired-end reads for NPB and 93–11 were generated with three biological replicates. Cleaned sequence reads for 30 samples were mapped against reference genome sequence for gene expression calculation (Additional file 1: Table S1). Differentially expressed genes (DEGs) were identified according to their significance in expression fold-change (false discovery rate (FDR) < 0.05 and at least two-fold change (|log2FC| > 1) in comparison to their respective control. The Pearson Correlation Coefficient of three biological replicates at three time points of rice cultivars infected by the two isolates are 0.96 (NPB) to 0.97 (93–11) (Additional file 1: Figure S3). We further validated the dataset obtained from RNA-seq with qRT-PCR by choosing 10 DEGs from NPB and 93–11 respectively. The correlation (R^2^) of RNA-seq and qRT-PCR results are 0.80 and 0.83 for NPB and 93–11 respectively, demonstrating high reliability of the sequencing results (Additional file 1: Figure S4). We also analyzed expression level of some important defense genes include *WRKY80, PR1a, PR1b, PR3, PR5, PR10, LOX, AOS, PAL* and *CHS1* in our RNA-seq data, and confirmed their up-regulation in our data (Additional file 1: Figure S5). Together, these results indicate high reproducibility of sequencing results.

### Comparative Expression Analysis of NPB and 93–11 Under *M. oryzae* Infection

**Fig. 1:**
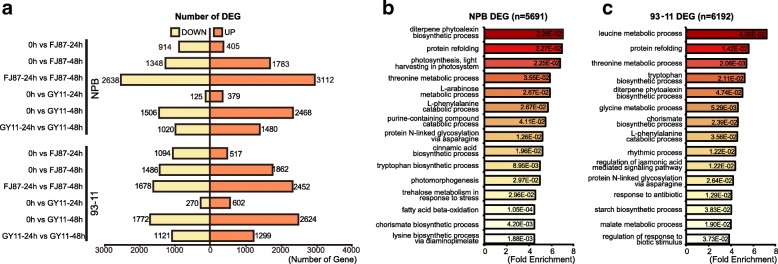
Number and GO enrichment of DEGs. **a** Up and down regulated genes in NPB and 93–11 between different time point and isolates. DOWN: down regulated genes. UP: up regulated genes. DEGs were defined as false discovery rate (FDR) < 0.05 and at least two-fold change (|log2FC| > 1). **b** and **c** Gene ontology (GO) enrichment of all differential expressed genes of NPB and 93–11. Fold Enrichment was calculated by observed gene number divided by expected gene number in each GO term. *P* value was calculated by Fisher’s Exact with Bonferroni correction.

A total of 27,844 (NPB) and 25,962 (93–11) genes were obtained and deployed for differential expression analysis after filtering-out lowly expressed genes. A total of 8,948 and 8,384 differentially expressed genes (DEGs) were identified in NPB and 93–11 by comparing between each other at different time points (Fig. 1a). As shown in Fig. 1a, with increased time of infection, the number of DEGs increased between mock control with 24 and 48 hpi in two rice cultivars, indicating more genes have been suppressed or activated during *M. oryzae* colonization. Moreover, though GY11 and FJ87 only exhibited slightly different aggressiveness on rice infection process, they can induce different response patterns on the two rice cultivars. For instance, we found that more DEGs of FJ87 infected rice than GY11 infected rice at 24 hpi, while more DEGs of GY11 infected rice than FJ87 infected rice at 48 hpi. In addition, we found that more DEGs between 24hpi and 48hpi in FJ87 inoculated NPB or 93–11 than GY11. It’s possible that these two isolates shown different fitness on two rice cultivars because these two isolates from distinct lineages, as we identified through large scale population genomic study, have some isolate-specific effectors that help pathogen manipulate plant early immunity activate different response genes in plant (Liao et al. 2016; Zhong et al. 2018).

To understand the response mechanisms to *M. oryzae* in two rice cultivars, we conducted Gene Ontology (GO) enrichment analysis of response genes in two rice cultivars. GO enrichment analysis revealed that numerous pathogen responsive pathways were activated during *M. oryzae* infection (Fig. 1b). For instance, diterpene phytoalexin biosynthetic pathway and chorismate biosynthetic pathway which have been shown to be activated in response to pathogen attack in various plants were enriched in DEGs carried-out in our study (Rao and Strange 1994; Wildermuth et al. 2001). In addition, we noticed genes belong to different GO term were enriched in two rice cultivars differently. For instance, trehalose metabolism, cinnamic acid processing and lysine biosynthetic process genes were enriched at NPB, while glycine metabolic process, regulation of jasmonic acid mediated signaling pathway and leucine metabolic process genes were enriched in 93–11, which suggest two rice cultivars may use different set of genes in response to *M. oryzae* infection (Bowles 1990; Schaff et al. 1995; Yang and Ludewig 2014; Zeier 2013).

### Differential Characteristics of Orthologous Genes in NPB and 93–11 in Response to Infection

NPB and 93–11 belong to *japonica* group and *indica* group respectively. These two rice groups divided several thousand years ago, which may have brought lots of differentiation in the genome (Sweeney and McCouch 2007). To determine the extent of divergence between two rice genomes, we compared genomic collinearity between the two cultivars. From this analysis, we observed the existence of high inter-genomic collinearity between the two cultivars. We also observed that, ~ 97.3% (13,501/13,876) of the orthologous genes showed high inter-genomic collinearity between NPB and 93–11. In addition, we further analyzed 13,876 of orthologous genes in 5 rice genomes (2 *japonica* and 3 *indica* rice), and found that these orthologous genes are also very conserved in these genomes (Additional file 1: Figure S6).

**Fig. 2:**
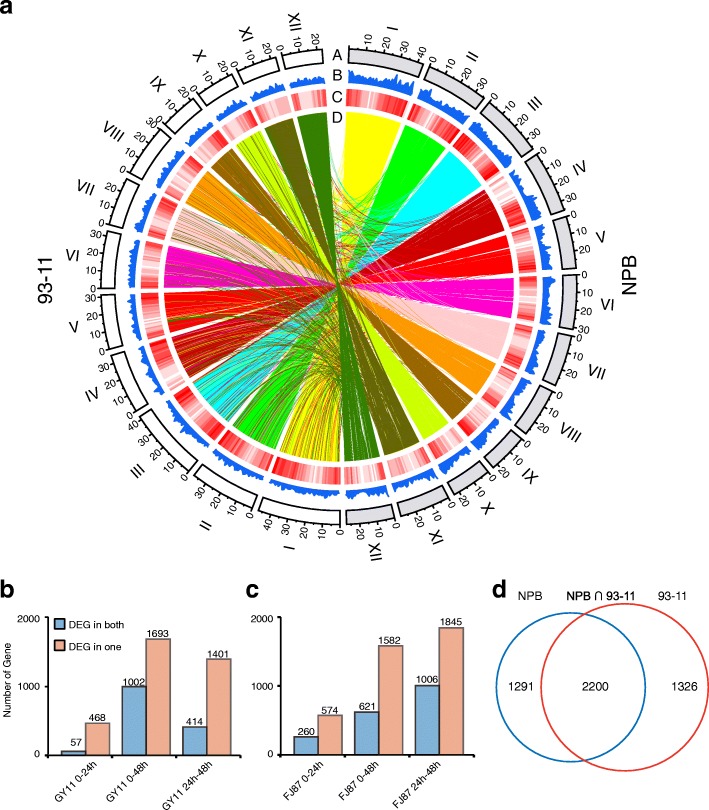
Different performance of orthologous genes in response to *M. oryzae* infection. **a** Circos map was used to describe whole genome composition, distribution of genes and collinearity of orthologous genes. Track a: 12 chromosomes of NPB and 93–11. Track b: numbers of unique genes in a 1 Mb window. Track c: density of orthologous genes over genome, white to red gradient color stands for dense and disperse regions respectively. Track d: collinearity of orthologous genes between two rice cultivars. **b** Number of orthologous DEGs in NPB and 93–11 in response to GY11 infection between different time points. **c** Number of orthologous DEGs in NPB and 93–11 in response to FJ87 infection between different time points. **d** Venn graph of DEGs orthologous genes. NPB: unique DEGs of NPB. 93–11: unique DEGs of 93–11. NPB ∩ 93–11: common DEGs of NPB and 93–11

To investigate whether NPB and 93–11 used different sets of genes in response to *M. oryzae* infection, we divided whole genome genes into 13,876 pairs of orthologous genes and 33,327 non-orthologous genes for further comparison. Interestingly, our studies showed that orthologous genes, which are considered as genetically conserved region in evolution, displayed differential expression profile in these two cultivars under infection (Table 1), indicating the existence of strong selection pressure on expression level of orthologous genes in the course of intensive domestication of *japonica* and *indica* rice subspecies as an evolutionary tool for resisting *M. oryzae* infection. Our investigations showed that a total of 3,491 (25.2%) and 3,526 (25.4%) of orthologous genes that exhibited differential expression pattern in the two rice cultivars at 24 hpi or 48 hpi, which is significantly higher than 19.4% and 17.4% of non-orthologs in NPB and 93–11 respectively (Table 1, two-sided Fisher’s exact test, *p*-value = 2.26e^− 42^ (NPB) and p-value = 2.73e^− 85^ (93–11)). By comparing orthologous DEGs number in two rice against GY11 or FJ87 at different time points, we found the number of DEGs in both NPB and 93–11 are much lower than DEGs at only one rice cultivar (either in NPB or 93–11) (Fig. 2b and c). To investigate overlapping of orthologous DEGs in the two rice, we merged DEGs at different time points or against different isolates and tested overlap between the two rice. As shown in the Venn diagram, 36.99% (1,291/3,491 for NPB) and 37.61% (1,326/3,526 for 93–11) of orthologous genes were differentially expressed in NPB and 93–11 at 24 hpi or 48 hpi of *M. oryzae* infection (Fig. 2d). From these results we inferred that, conserved orthologous genes identified in the two cultivars evolved independently from each other and rice blast pathogens evolved different strategies to adapt to the host basal resistance.

**Table 1:** Rates of orthologous DEGs and non-orthologous DEGs in 93–11 and NPB in response to *M. oryzae* infection

	NPB				93–11			
	Expressed	Rate	DEGs	Rate	Expressed	Rate	DEGs	Rate
Ortholog	10,337	74.5%	3,491	25.2%	10,142	73.1%	3,526	25.4%
Non-Ortholog	17,507	62.0%	5,485	19.4%	15,820	56.0%	4,907	17.4%
Total	27,844	66.1%	8,948	21.2%	25,962	61.6%	8,384	19.9%

### Variation in Promoter Region May Account for Divergence in the Expression Pattern of Orthologous Genes

**Fig. 3:**
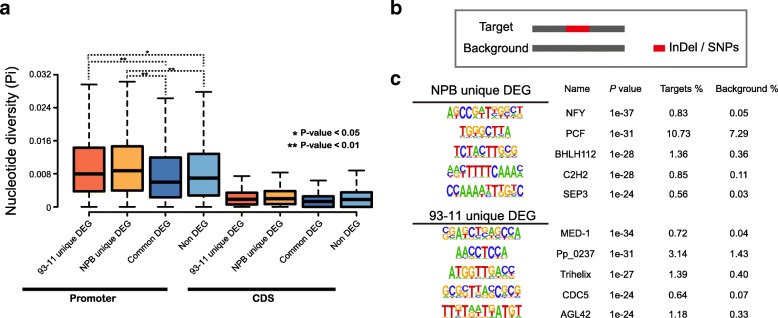
High differentiation at promoter region**. a** Boxplot showing nucleotide diversity (Pi) of 93–11 unique DEGs, NPB unique DEGs, common DEGs and Non DEGs at promoter and CDS regions. * represents *p*-value smaller than 0.05, ** represents p-value smaller than 0.01 estimated with student’s t-test. **b** Schematic of sequences used in motif enrichment analysis. Target sequences are sequences that have an InDels or SNPs with 25 bp up/down-stream flanking sequence. Background sequences are corresponding site at opposite rice that have no InDels or SNPs but only 25 bp up/down-stream flanking sequence. **c** Top 10 enriched transcription factor binding motif in promoter regions that have InDels or SNPs in NPB or 93–11

Although orthologous genes showed high genomic collinearity and sequence similarity (> 95%), they however responded differently to biotic stress induced by *M. oryzae* infection. We speculated promoter regions, where the *cis*-regulatory elements are enriched, may have experienced significant changes. To compare variation level of promoter regions, we divided orthologous genes into NPB and 93–11 common DEGs (common DEG), NPB specific DEGs (NPB unique DEG), 93–11 specific DEGs (93–11 unique DEG) and the rest that not DEG in NPB or 93–11 (Non DEG), and compared their nucleotide diversity in both promoter regions and coding sequence (CDS). Since the sequence similarity level of orthologous genes in two rice will greatly affect sequence alignment as well as nucleotide diversity calculation, we decided to use more stringent methods to filter out less conserved orthologous genes. To achieve this, only genes with similar expression level and high sequence similarity between two rice were defined as common DEGs. Consistently, only genes that have differential expression level between two cultivars and high sequence similarity were defined as NPB/93–11 unique DEGs. Finally, 547 and 629 pairs of unique DEGs of NPB and 93–11 as well as 1,222 pairs of common DEGs, 9,095 pairs of non DEGs were identified. By comparing nucleotide diversity of these four groups, we found that nucleotide diversity in promoter regions is generally higher than that of CDS and as expected, NPB/93–11 unique DEGs exhibited significantly higher nucleotide diversity than common DEGs or Non DEGs (Student’s *t*-test, *p*-value equal to 7.89e^-05^ in 93–11 unique DEGs group and 1.21e^-05^ in NPB unique DEGs group). As shown in Fig. 3a, average level of nucleotide diversity for 93–11 unique DEGs, NPB unique DEGs, common DEGs and non DEGs at promoter regions are 0.0136, 0.0144, 0.0106 and 0.0121, compared with 0.003, 0.004, 0.002 and 0.003 at CDS regions respectively. In addition, average level of nucleotide diversity for 93–11 unique DEGs, NPB unique DEGs are significantly higher than that of common DEGs and non DEGs at promoter regions (0.0136 and 0.0144 vs. 0.0106 and 0.0121).

NPB unique DEGs or 93–11 unique DEGs are genes that specifically and differentially expressed in NPB or 93–11, which may be mediated by variation at promoter region. To investigate whether variation at promoter regions results in *cis*-regulatory elements variation, we set out to conduct *cis*-regulatory elements enrichment of these regions. To achieve this, we collected sequences at promoter regions with SNPs or InDel between NPB and 93–11 and its 25 bp up/down flanking sequences and applied to motif enrichment analysis with the same regions of orthologs at opposite rice cultivar as background control (Fig. 3b). From this analysis, we have identified 29,921 SNPs and 10,135 InDels at promoter regions of NPB/93–11 unique DEGs in the two rice cultivars. Motif enrichment analysis indicated that promoter sequences variation lead to *cis*-regulatory elements variation. Figure 3c listed out top 10 most enriched transcription factor binding motif in NPB and 93–11. Among these *cis*-regulatory elements variation, NFY, PCF, BHLH112, a C2H2 TF and SEP3 are of the most enriched transcription factor binding motif in NPB. While MED-1, Pp_0237, Trihelix, CDC5 and AGL42 binding site are the most abundant transcription factor binding motif identified in promoter regions of 93–11 unique DEGs.

### Variation in Promoter Regions is Associated with Rice Divergence

**Fig. 4:**
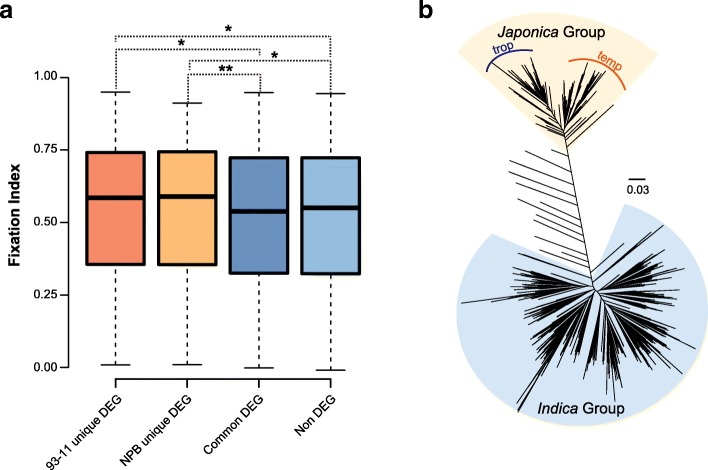
Rice cultivar unique DEGs have higher Fst. **a** Boxplot showing fixation index (Fst) of 93–11 unique DEG, NPB unique DEG, common DEG and Non DEG at promoter region. * represents p-value smaller than 0.05, ** represents p-value smaller than 0.01 with student’s t-test. **b** Phylogenetic tree of *japonica* and *indica* group based on SNPs/InDels in NPB and 93–11 unique DEGs. Trop: tropical *japonica* rice. Temp: temperate *japonica* rice

After several thousand years of domestication, *japonica* and *indica* rice constitutes two largest rice subspecies in cultivated rice, during which most important traits such as seed dormancy, shattering, seeds number, seed size and resistance have been fixed in the population (Sweeney and McCouch 2007). Since rice blast disease is one of the major biotic stress to rice, we hypothesized traits that relate to *M. oryzae* response also experienced strong selection during rice domestication. Thus, our next question is to ask whether the expression divergence mediated by *cis*-elements variation is also involved in the population divergence of rice *japonica* and *indica* groups. To test this hypothesis, we analyzed fixation index (Fst) of identified variation sites in population data of previously published 1,770 *indica* and 850 *japonica* rice accessions, which represents global *japonica* and *indica* rice cultivars. In consistent with former study, the average Fst level of selected sites between *japonica* and *indica* groups is 0.526 (Fig. 4a), which indicate a strong differentiation between two rice subspecies (Huang et al. 2010). However, Fst value at promoter regions containing *cis*-elements variation in NPB and 93–11 unique DEGs are 0.547 and 0.541 respectively, which is significantly higher than that of common DEG (Fst = 0.518) and non DEGs (Fst = 0.523). In line with phylogenetic tree based on whole genome SNPs data in previous study, the phylogenetic tree based on SNPs/InDels in NPB and 93–11 unique DEGs also showed that *japonica* and *indica* rice groups could be divided into two well-defined clades (Fig. 4b), in which *japonica* group can be further defined as tropical *japonica* rice (trop) and temperate *japonica* rice (temp) (Wang et al. 2018b).

## Discussion

Interplay between environmental factors and diverse genomic features enable various rice cultivars to possess firm resistance against invading pathogenic microbes and also equip them with the requisite traits to thrive under harsh conditions (López-Arredondo et al. 2015). Although researches have shown that different rice subspecies exhibit differential resistance against the blast fungus *M. oryzae* (Gallet et al. 2016), details regarding genomic parameters accounting for the differential response of different rice subspecies to blast infection has not been extensively studied. This study provides an insight into how variation in the promoter region of orthologous genes contribute to the differential response of two rice cultivars, NPB and 93–11 each from *japonica* and *indica* subspecies during *M. oryzae* infection. The short divergence time between *japonica* and *indica* rice as well as high quality genome sequences makes these two rice cultivars good models in solving molecular, genetic and evolutionary questions (He et al. 2011; Liao et al. 2016; Tang et al. 2006). We identified 13,876 pairs of orthologous between NPB and 93–11 genomes, and most of these genes exhibit similar or identical chromosome location in the genomic collinear analysis. However, our comparison revealed significant difference in the expression of orthologous genes identified in the two cultivars during *M. oryzae* infection. Since orthologous genes are considered as highly conserved throughout divergence history of the two *japonica* and *indica* rice cultivars (Sweeney and McCouch 2007), we opine that the observed emerging divergence in the expression pattern of orthologous genes between NPB and 93–11 rice cultivars challenged with *M. oryzae* could be an individually acquired evolutionary adaptation developed by the two rice cultivars in an attempt to durably resist rice blast fungus.

It was proposed that gene expression divergence related with adaptive phenotypes may lead to speciation (Haerty and Singh 2006). With the development of next generation sequencing, accumulating evidences have been reported showing expression divergence of orthologous genes between closely related species in response to various stresses. Expression divergence between pine and spruce, which divergent ~ 140 million years ago, is important for survival in the face of climatic change (Yeaman et al. 2014), and this phenomenon is also allowing domesticated and wild tomato tackle wound stress (Liu et al. 2018). In our study, we observed high incidence of variation in promoter region of orthologous genes that are known to be enriched with gene regulatory elements. We deduced that inter-cultivar variations observed in the expression pattern of orthologous genes are as a result of nucleotide diversities associated with the promoter region sequences of orthologous genes from the two cultivars. This hypothesis is supported by previous researches which showed that mutation in the *cis*-elements of individual promoter could cause phenotypic variation (Hoffmann et al. 2016; Xing et al. 2008). Furthermore, our studies also identified genome-wide correlation between variations occurring in the promoter region of *M. oryzae* infection responsive genes and their transcriptional divergence between NPB and 93–11.

Extensive studies indicated that sequence variation in promoter regions of *cis*-regulatory elements affects the specificity or binding activity of Transcription Factor (TF), which subsequently affects expression pattern of its proximal genes and resulted in obvious phenotypic changes. For instance, a single SNP at *bsr-d1* promoter enhances binding to MYBS1 and promote broad-spectrum rice blast disease resistance in rice (Li et al. 2017). Small insertions in *ARE1* promoter in *indica* and *aus* accessions enhance grain yield under nitrogen-limiting conditions (Wang et al. 2018a). We inferred that, high variation in promoter region may trigger difference in the binding efficiency of TFs and hence, resulting in expression divergence of infection responsive genes in the two rice cultivars investigated in this study (Tran et al. 2004; Yoshida et al. 1998). Consistent with this hypothesis, we found TF binding motif of NFY *CCAAT* box, which is important for drought response in maize (Nelson et al. 2007), were enriched specifically at promoter regions variation sites of NPB unique DEGs. TF binding motif of PCF (also named TEOSINTE-BRANCHED1, TCP), which plays key roles in plant systemic acquired resistance, was also enriched among promoter of NPB unique DEGs (Li et al. 2018). BHLH112 is characterized as abiotic stress tolerance in *Arabidopsis* (Liu et al. 2015). In addition, GATA factor-related transcription factors MED-1, CDC5 and AGL42 enriched at promoter variation sites of 93–11 unique DEGs are also reported to be involved in development and immunity (Broitman-Maduro et al. 2005; Nawy et al. 2005; Palma et al. 2007). The population genomic analysis in our study further indicated that variations that affected TF binding activity may subsequently lead to phenotypic changes between *japonica* and *indica* rice in response to biotic stress and had been gradually fixed in population during domestication.

## Conclusions

In summary, our genome-wide comparative analysis showed that, higher sequence diversity in promoter region is tightly associated with expression divergence of evolutionarily conserved orthologous genes in NPB and 93–11 cultivars, which may subsequently account for their differential response to *M. oryzae* infection. We proposed that variation in the expression pattern of orthologous genes in two rice cultivars may act as an evolutionary alternative distinguishing the two genetically diverse rice subspecies response to *M. oryzae* infection. Genetic variations among the two rice subspecies identified in this study also provide valuable resource that could be explored as the target for genetic engineering research.

## Additional file

Additional file 1: **Table S1.** Total number of sequencing reads and mapping results for 30 samples in this study. **Table S2.** qRT-PCR primers for RNA-seq confirmation used in this study. **Figure S1.** Infection assay of GY11 and FJ87 on NPB and 93-11. **Figure S2.** Workflow of rice inoculation and samples collections. **Figure S3.** Pearson Correlation Coefficient between sequenced samples. **Figure S4.** Correlation (R^2^) of 10 DEGs expression between RNA-seq and qRT-PCR results. **Figure S5.** Expression level of rice defense genes in RNA-seq. **Figure S6.** Number of conserved orthologous genes in 5 rice genomes. (DOCX 2481 kb)

## Abbreviations

bp: Base Pair
DEG: Differentially expressed genes
FDR: False discovery rate
FJ87: FJ81278
FPKM: Fragments Per Kilobase of transcript per Million mapped reads
Fst: Fixation index
GO: Gene Ontology
GY11: Guy11
hpi: Hours Post Inoculation
NPB: Nipponbare
nt: Single-stranded DNA/RNA units of nucleotides
qRT-PCR: Quantitative Real Time-PCR
QTL: Quantitative Trait Locus
SNP: Single-nucleotide polymorphism
TF: Transcription factor
