# Supplementary material for: Expression Divergence as an Evolutionary Alternative Mechanism Adopted by Two Rice Subspecies Against Rice Blast Infection
Source: Rice (N Y). 2019 Mar 1;12:12. doi: 10.1186/s12284-019-0270-5 (PMC6397267; doi:10.1186/s12284-019-0270-5)
Supplement: Supplementary file 1 — Table S1. Total number of sequencing reads and mapping results for 30 samples in this study. Table S2. qRT-PCR primers for RNA-seq confirmation used in this study. Figure S1. Infection assay of GY11 and FJ87 on NPB and 93-11. Figure S2. Workflow of rice inoculation and samples collections. Figure S3. Pearson Correlation Coefficient between sequenced samples. Figure S4. Correlation (R2) of 10 DEGs expression between RNA-seq and qRT-PCR results. Figure S5. Expression level of rice defense genes in RNA-seq. Figure S6. Number of conserved orthologous genes in 5 rice genomes. (DOCX 2481 kb) [file 12284_2019_270_MOESM1_ESM.docx]

**Table S1** Total number of sequencing reads and mapping results for 30 samples in this study.

| **Sample** | **NPB** | | | | |  | | | **93-11** | | | | |
| --- | --- | --- | --- | --- | --- | --- | --- | --- | --- | --- | --- | --- | --- |
|  | **Total reads** | **Aligned pairs** | **Aligned Left** | **Aligned Right** | **Mapping rate** | |  | **Total reads** | | **Aligned pairs** | **Aligned Left** | **Aligned Right** | **Mapping rate** |
| 0h-1 | 27542910 | 11724903 | 12901992 | 12088257 | 93.69% | |  | 27401242 | | 10026907 | 11623545 | 10876522 | 82.11% |
| 0h-2 | 29544202 | 12433773 | 13768566 | 12814396 | 93.21% | |  | 25733522 | | 9396485 | 10948553 | 10215825 | 82.24% |
| 0h-3 | 27125594 | 11596697 | 12759735 | 11931426 | 94.08% | |  | 27040236 | | 10110147 | 11627675 | 10907429 | 83.34% |
| GY11_24h-1 | 24832370 | 10658967 | 11698823 | 10966267 | 94.22% | |  | 34906380 | | 12931565 | 14952318 | 13985001 | 82.90% |
| GY11_24h-2 | 23362546 | 9812281 | 10972016 | 10094843 | 93.93% | |  | 27918664 | | 10275477 | 11957942 | 11145041 | 82.75% |
| GY11_24h-3 | 18249940 | 7707318 | 8556347 | 7938855 | 93.77% | |  | 29545662 | | 10874766 | 12681223 | 11780119 | 82.79% |
| GY11_48h-1 | 30169068 | 12846938 | 14154446 | 13265418 | 93.83% | |  | 34923192 | | 13055350 | 14978587 | 14054950 | 83.14% |
| GY11_48h-2 | 36243644 | 15461083 | 17014900 | 15966175 | 93.89% | |  | 31039058 | | 11221595 | 13192166 | 12154435 | 81.66% |
| GY11_48h-3 | 31123250 | 13337085 | 14681719 | 13747510 | 94.35% | |  | 32668548 | | 11911109 | 13898833 | 12959796 | 82.22% |
| FJ87_24h-1 | 24676158 | 10450435 | 11533414 | 10792893 | 93.48% | |  | 21018256 | | 7799863 | 8973241 | 8411984 | 82.71% |
| FJ87_24h-2 | 21792536 | 9277409 | 10163320 | 9595335 | 93.27% | |  | 29589828 | | 10923628 | 12645546 | 11821871 | 82.69% |
| FJ87_24h-3 | 27360302 | 11643117 | 12819465 | 12010763 | 93.71% | |  | 23832440 | | 8650161 | 10182734 | 9351761 | 81.97% |
| FJ87_48h-1 | 26423130 | 11196614 | 12434852 | 11528078 | 94.12% | |  | 29465324 | | 10981929 | 12703037 | 11790333 | 83.13% |
| FJ87_48h-2 | 30335924 | 12906644 | 14273196 | 13294140 | 94.10% | |  | 29000498 | | 10764684 | 12449980 | 11630677 | 83.04% |
| FJ87_48h-3 | 23366356 | 9995183 | 10979818 | 10290574 | 93.98% | |  | 29936348 | | 10845130 | 12859680 | 11704889 | 82.06% |

**Table S2** qRT-PCR primers for RNA-seq confirmation used in this study.

| Gene ID | Description | Primer | Sequence (5' -> 3') |
| --- | --- | --- | --- |
| Os-actin | Actin gene | Forward | GTTACTCATTCACCACAACGGC |
|  |  | Reverse | CCTTTCAGGAGGGGCGACC |
| BGIOSGA037068 | Citrate-binding protein | Forward | CCAGCTCACCGTCTTCATT |
|  |  | Reverse | TACACGCCGAACTTGAAGTAG |
| BGIOSGA006958 | Lipoxygenase 2.3 | Forward | ACTTCGGGCAGTACCACTA |
|  |  | Reverse | CTTCATCACCTCCTCCTTGTTC |
| BGIOSGA002618 | Transcription factor bHLH168 | Forward | GCGTGCTGGAGGAAGAAG |
|  |  | Reverse | CTGGGAGTGGAGAGTGTAGAA |
| BGIOSGA002483 | Bowman-Birk type bran trypsin inhibitor | Forward | TTGCAACGACGAGGTGAA |
|  |  | Reverse | CGCAGACGAAGACGACAT |
| BGIOSGA011103 | Uncharacterized | Forward | CGAAGCCAGCAAGGTTAATG |
|  |  | Reverse | ACACAAACTACAGCGGAGAC |
| BGIOSGA031578 | Pectinesterase inhibitor 7 | Forward | CGACAACATGTGCATGGAAG |
|  |  | Reverse | TGGAGGAGGCCCATGAT |
| BGIOSGA005998 | Phenylalanine ammonia-lyase-like | Forward | CGTCAACTCTCTTGGACTCATC |
|  |  | Reverse | GATCAAGAACGTGGAGGACAT |
| BGIOSGA005869 | SK3-type dehydrin | Forward | GCTGCTCCTGCCACTAC |
|  |  | Reverse | CTTGCCCAGTATACCCTTCTTC |
| BGIOSGA022314 | GDSL esterase/lipase APG | Forward | CTCGCTTTCGAAGACCTACTC |
|  |  | Reverse | GAATCCTTGGGATTGAGGAGAG |
| BGIOSGA024205 | Thaumatin-like protein | Forward | CTGTCGTGCAGGCTGTC |
|  |  | Reverse | GGTTGTAGAAGTCCCGGTTG |
| OS12G0170800 | Citrate-binding protein | Forward | CAGCCGGAGCCGATAAATAA |
|  |  | Reverse | CCGTGAAATGGAATGAGCAAAC |
| OS02G0194700 | Lipoxygenase 2.3 | Forward | GAGGCATTTGCTAGTGTGTTTG |
|  |  | Reverse | ACTCAGCGAACTGAGCTTTAG |
| OS01G0108600 | Transcription factor bHLH168 | Forward | GCGGGAGCAAGCTGTTATTA |
|  |  | Reverse | CTCCTACATGATAGACTGGAGACA |
| OS01G0124000 | Bowman-Birk type bran trypsin inhibitor | Forward | GATCAGTCTGCGTGTGTTCT |
|  |  | Reverse | CACGCATACCAACATCAAACC |
| OS03G0251000 | Uncharacterized | Forward | GCTCTCGTCAATCTGAAGGTC |
|  |  | Reverse | ACGCTATCTCTCGACGTACTTA |
| OS12G0630100 | Thaumatin-like protein | Forward | CAACGGCAACAGCAACTAC |
|  |  | Reverse | TTCCCTGATACAGTTTAGTAGAAGT |
| OS02G0627100 | Phenylalanine ammonia-lyase-like | Forward | TGGCTATCAACGAAGGCAAG |
|  |  | Reverse | CCTCCACACTCCACTGTTATTC |
| OS10G0508700 | Pectinesterase inhibitor 7 | Forward | GCACAGACTGAAGTTGATTTGG |
|  |  | Reverse | TGTTGTGCTCCTGTGGTTTA |
| OS02G0669100 | Dehydrin DHN1-like | Forward | CAGGCCATGGTTGGAATTTG |
|  |  | Reverse | GACCAATCTTGACACGAACTTAAC |
| OS06G0148200 | GDSL esterase/lipase APG | Forward | CAGAGGGCTGCATTGTAGAT |
|  |  | Reverse | CAAACCACCGAGCAAACATTAT |


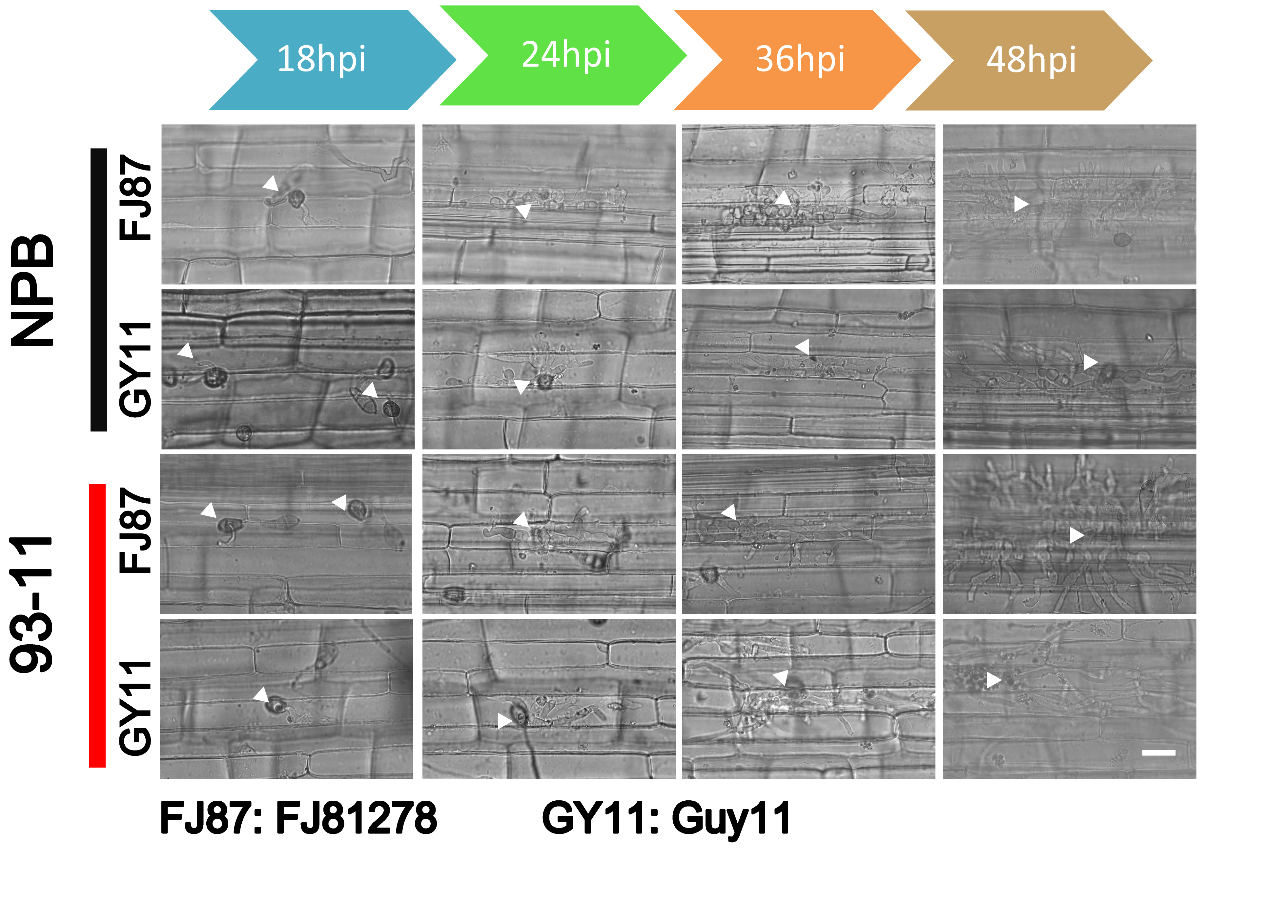


**Figure S1** **Infection assay of GY11 and FJ87 on NPB and 93-11.** Rice sheath of NPB and 93-11 have been inoculated by *M. oryzae* isolates, Guy11 (GY11) and FJ81278 (FJ87) and observed at 18, 24, 36, 48 hours post inoculation (hpi). Bar=20㎛. White triangle=infection site.


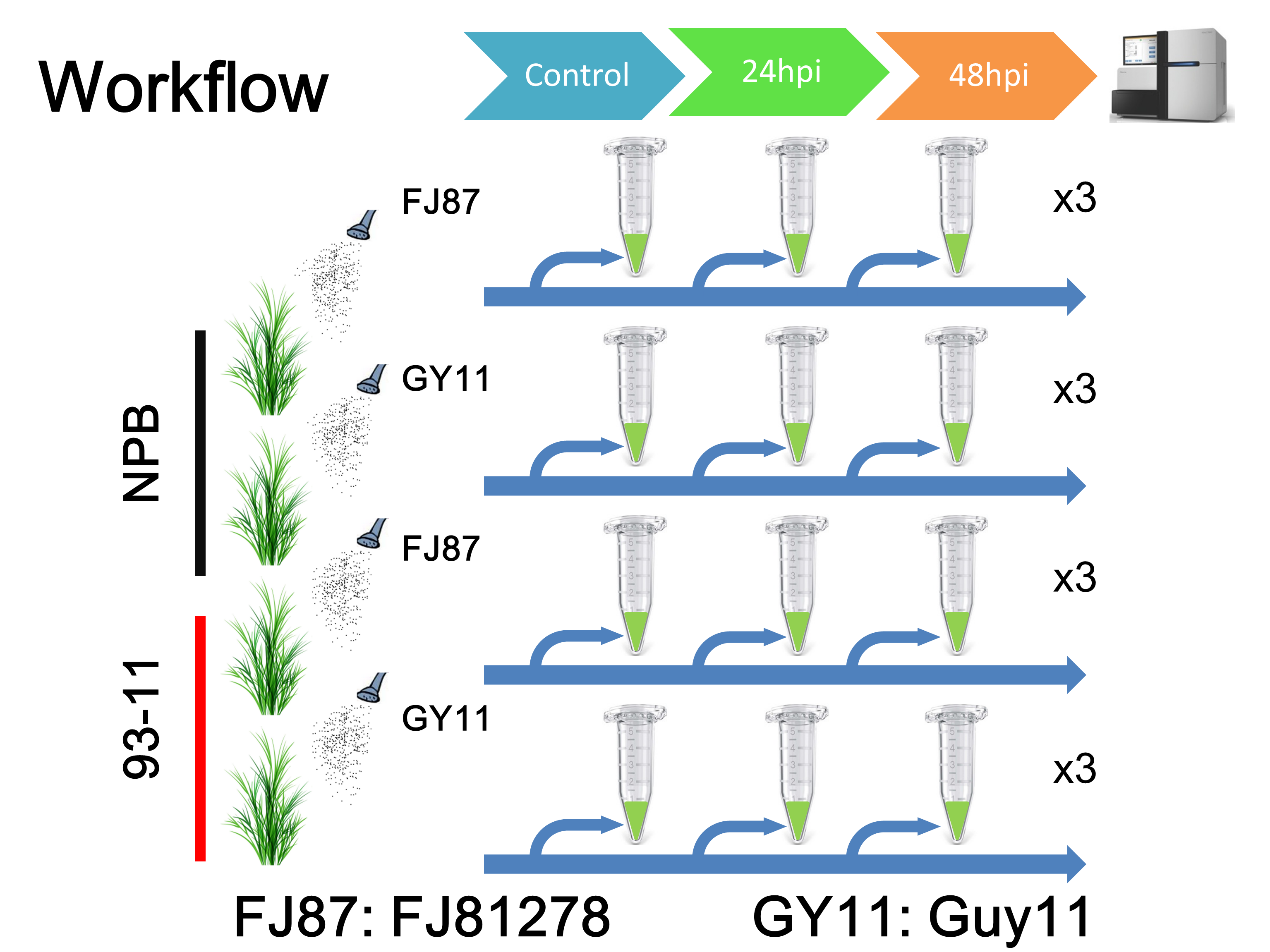


**Figure S2** **Workflow of rice inoculation and samples collections.** The *japonica* cultivar NPB and the *indica* cultivar 93-11 have been used in our study. *M. oryzae* isolates, Guy11 (GY11) and FJ81278 (FJ87), charactized as isolates belonging to two distinguished lineages have been used for inoculation with three biological replicates.


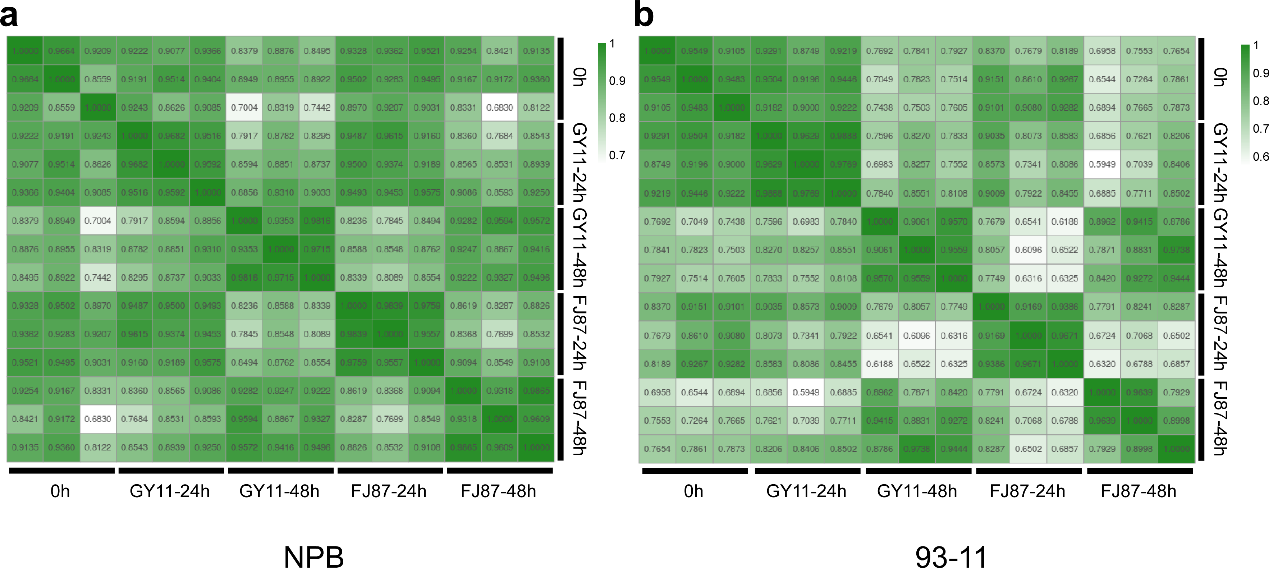
**Figure S3 Pearson Correlation Coefficient between sequenced samples.** **a.** Pearson Correlation Coefficient of NPB samples. **b.** Pearson Correlation Coefficient of 93-11 samples.


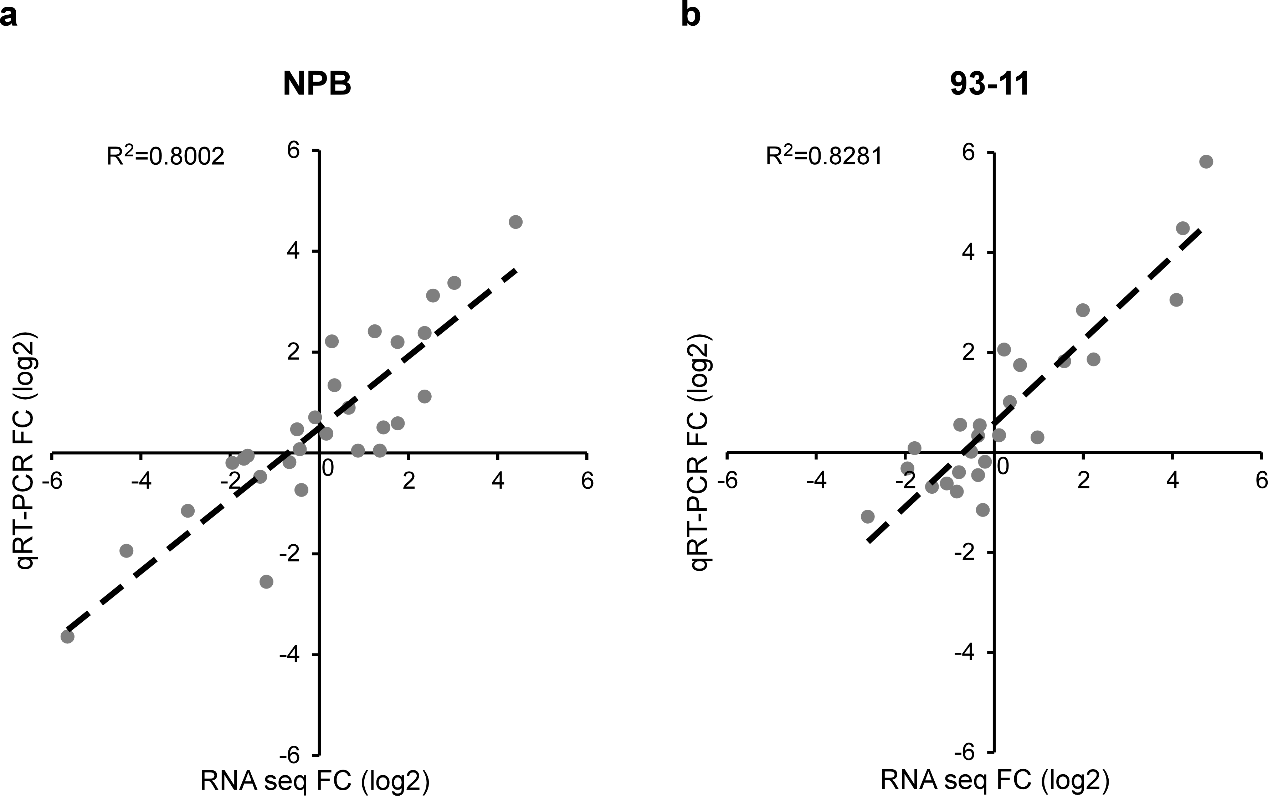
**Figure S4** **Correlation (R^2^) of 10 DEGs expression between RNA-seq and qRT-PCR results.** Correlation (R2) of 10 DEGs expression between RNA-seq and qRT-PCR results for NPB **(a)** and 93-11 **(b)**. Relative expression level for RNA-seq and qRT-PCR results were plot on y and x axis. qRT-PCR primers used in this study are presented in the **Supplementary Table 2.**


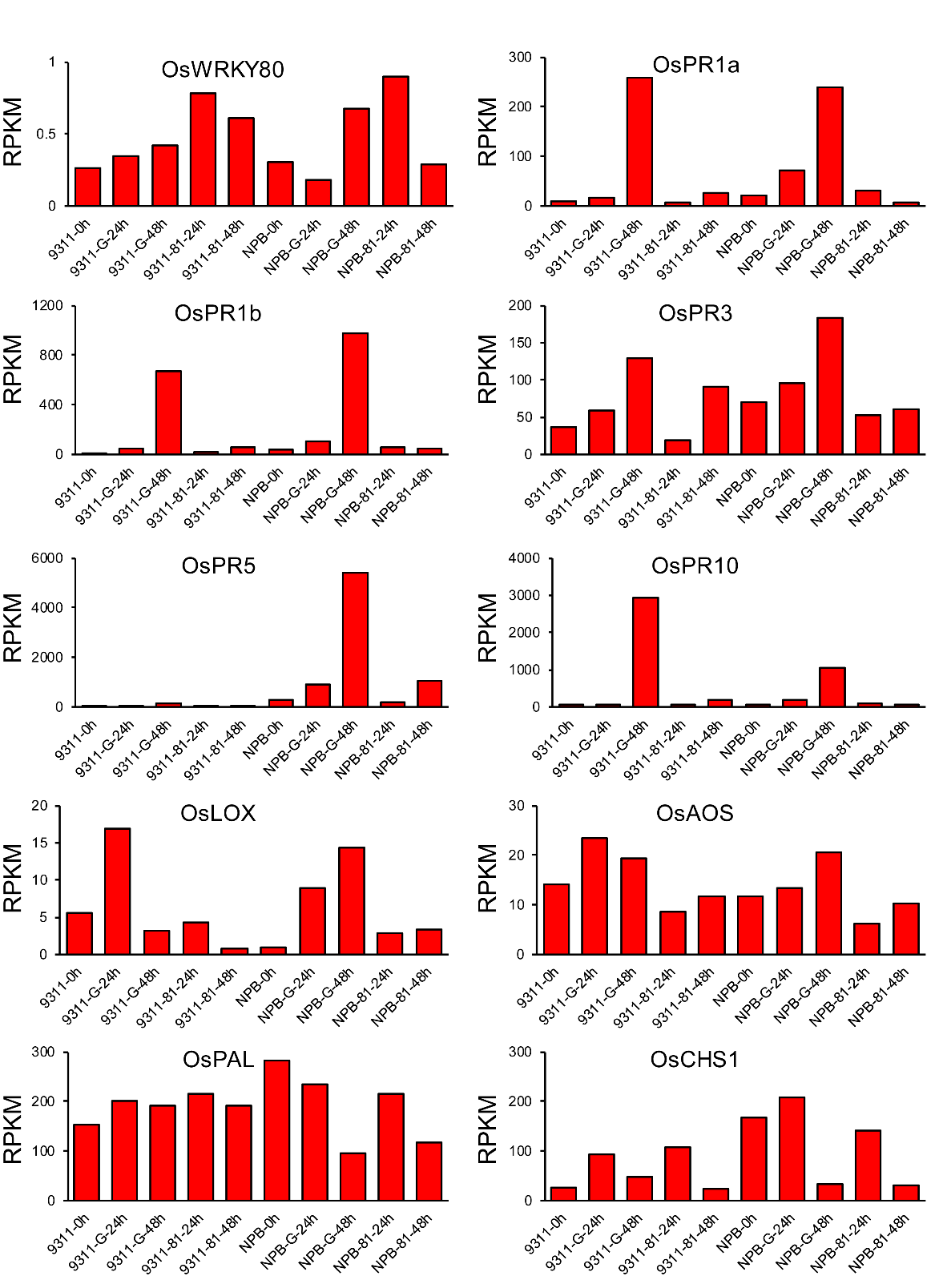


**Figure S5 Expression level of rice defense genes in RNA-seq.** Expression level of rice defense gene; *WRKY80, PR1a, PR1b, PR3, PR5, PR10, LOX, AOS, PAL* and *CHS1* in RNA-seq. Expression level is present with RPKM (Reads Per Kilobase Million).


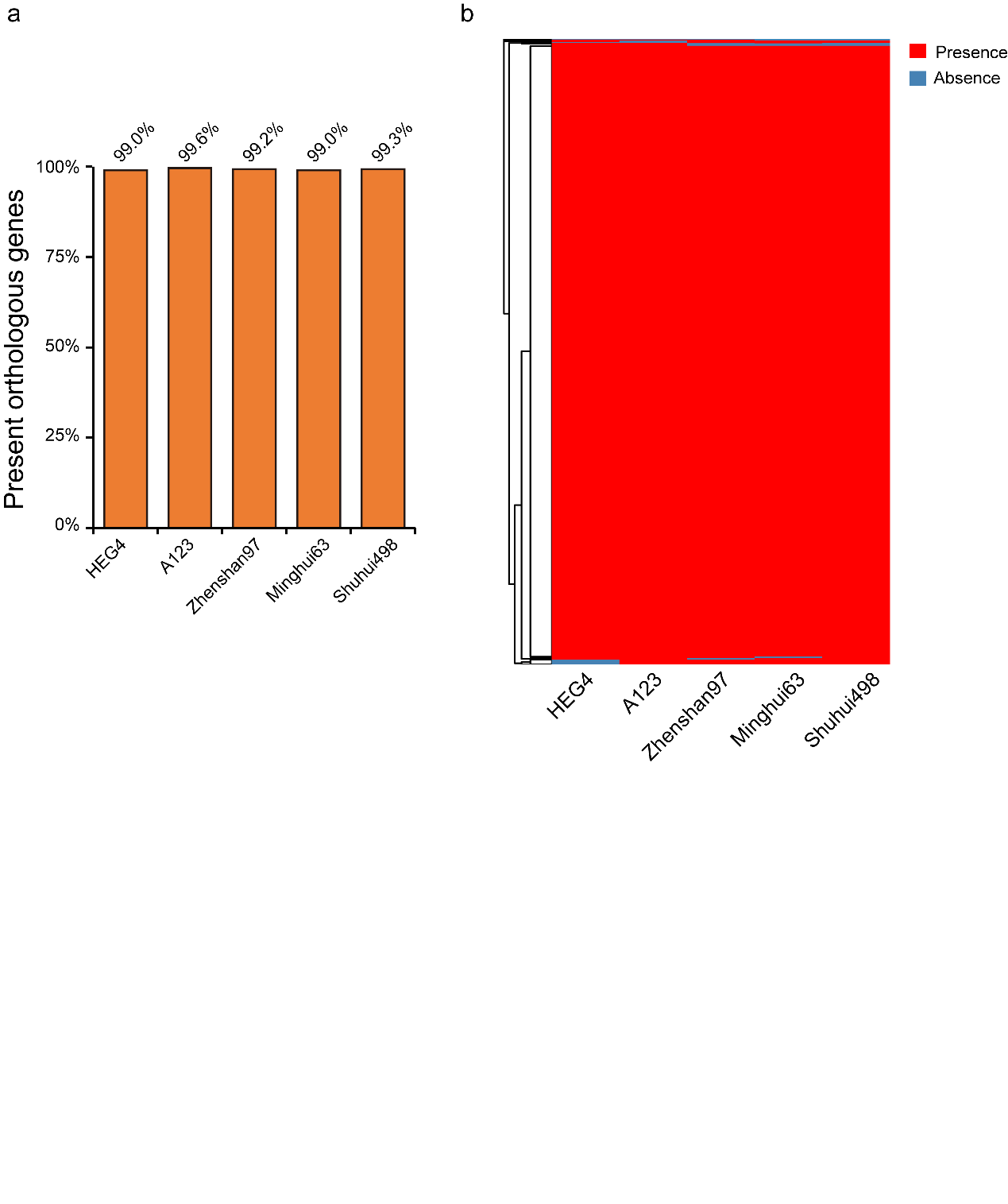


**Figure S6 Number of conserved orthologous genes in 5 rice genomes.** a Percentage of conserved orthologous genes (n=13,876) in 3 indica rice (Zhenshan 97, Minghui 63, Shuhui 498), 2 japonica rice (HEG4, A123). b Heatmap of present and absent polymorphism of conserved orthologous genes (n=13,876) in 3 indica rice (Zhenshan 97, Minghui 63, Shuhui 498), 2 japonica rice (HEG4, A123).
